# Use of Tissue Expander and Breast Implant in Postmastectomy Reconstruction: Presentation of a Clinical Case

**DOI:** 10.7759/cureus.84168

**Published:** 2025-05-15

**Authors:** Arym P Preza Estrada, Gladys M Ballesteros Solís, Harvey Y Zamora-Veliz, José L Villarreal-Salgado, Gerardo S Rea-Martínez

**Affiliations:** 1 General Surgery, Instituto de Seguridad y Servicios Sociales de los Trabajadores del Estado (ISSSTE) "General Hospital", La Paz, MEX; 2 General Surgery, "Fray Antonio Alcalde" Civil Hospital, Guadalajara, MEX; 3 General Surgery, Instituto de Seguridad y Servicios Sociales de los Trabajadores del Estado (ISSSTE) "Clinic Hospital", Mazatlan, MEX; 4 Plastic and Reconstructive Surgery, Instituto de Seguridad y Servicios Sociales de los Trabajadores del Estado (ISSSTE) "Valentín Gómez Farías Regional Hospital", Zapopan, MEX

**Keywords:** breast cancer, breast reconstruction, plastic surgery, postmastectomy, tissue expanders

## Abstract

Breast cancer remains the most common malignancy among women worldwide, and mastectomy continues to be a standard treatment for many patients. Advances in oncological therapies have led to increased survival rates, emphasizing the need for reconstructive options that not only restore physical anatomy but also improve psychological well-being and overall quality of life. Breast reconstruction using tissue expanders has become a widely accepted technique, offering a staged approach that allows gradual accommodation of an implant while preserving the skin and soft tissue envelope. This method is particularly beneficial in cases where autologous tissue is insufficient or when patients prefer a less invasive initial procedure. Technological innovations have led to the development of anatomically shaped expanders and integrated valve systems, enhancing both safety and aesthetic outcomes. Despite its advantages, expander-based reconstruction is not without complications, with risks such as infection, extrusion, capsular contracture, and delayed wound healing, particularly in patients undergoing adjuvant radiotherapy. Successful outcomes depend largely on appropriate patient selection, meticulous surgical technique, and careful postoperative management. The use of acellular dermal matrices and advances in expander design have further expanded the possibilities for optimizing results. Tissue expander breast reconstruction remains a safe, effective, and customizable option for many breast cancer survivors. As research progresses, future innovations in biomaterials, surgical techniques, and adjuvant therapies are expected to continue improving the durability, safety, and aesthetic satisfaction of reconstructive outcomes, ultimately contributing to better long-term physical and psychological recovery for patients following breast cancer treatment.

## Introduction

Breast cancer is one of the most common cancers among women worldwide, and its comprehensive treatment often includes mastectomy as a therapeutic or preventive measure. Given this situation, breast reconstruction has become a fundamental pillar in the physical and emotional recovery of patients, offering not only anatomical restoration but also a significant improvement in their quality of life and self-esteem [1].

The selection of the breast reconstruction method should be tailored to each patient, considering various factors such as current oncological status, individual anatomical characteristics, the need for radiation therapy, and personal aesthetic expectations. The most commonly used techniques include reconstruction with prosthetic devices (tissue expanders and implants), autologous flap procedures such as transverse rectus abdominis muscle (TRAM) flaps, deep inferior epigastric perforator (DIEP) flap, or latissimus dorsi flap, as well as hybrid approaches that combine both modalities.

Now, the trend has evolved toward less invasive techniques with more natural results, lower morbidity, and reduced surgical times. Innovations such as the use of acellular dermal matrices, the prepectoral approach, autologous fat grafting, and microsurgical techniques have expanded the reconstructive possibilities.

This review article aims to analyze the main surgical techniques used in postmastectomy breast reconstruction and their indications, benefits, and limitations, based on the most current evidence available.

## Case presentation

We present the case of a 56-year-old female patient with a personal history of systemic arterial hypertension and type 2 diabetes mellitus. Four years ago, she was diagnosed with invasive ductal adenocarcinoma breast cancer, with metastasis to the left mammary gland. The immunohistochemical profile showed positive estrogen receptor (+++), positive progesterone receptor, and negative Her2/neu. The patient denies a family history of cancer, as well as tobacco and alcohol use. Her gynecological and obstetric history includes two cesarean sections, the last performed 27 years ago.

As part of her oncological treatment, she received five cycles of chemotherapy and 25 sessions of radiotherapy. She subsequently underwent a left radical mastectomy, followed by breast reconstruction with a latissimus dorsi flap and tissue expander placement in 2024. During 2024 and 2025, the expander was inflated monthly with saline solution until reaching a final volume of 375 cc.

**Figure 1:**
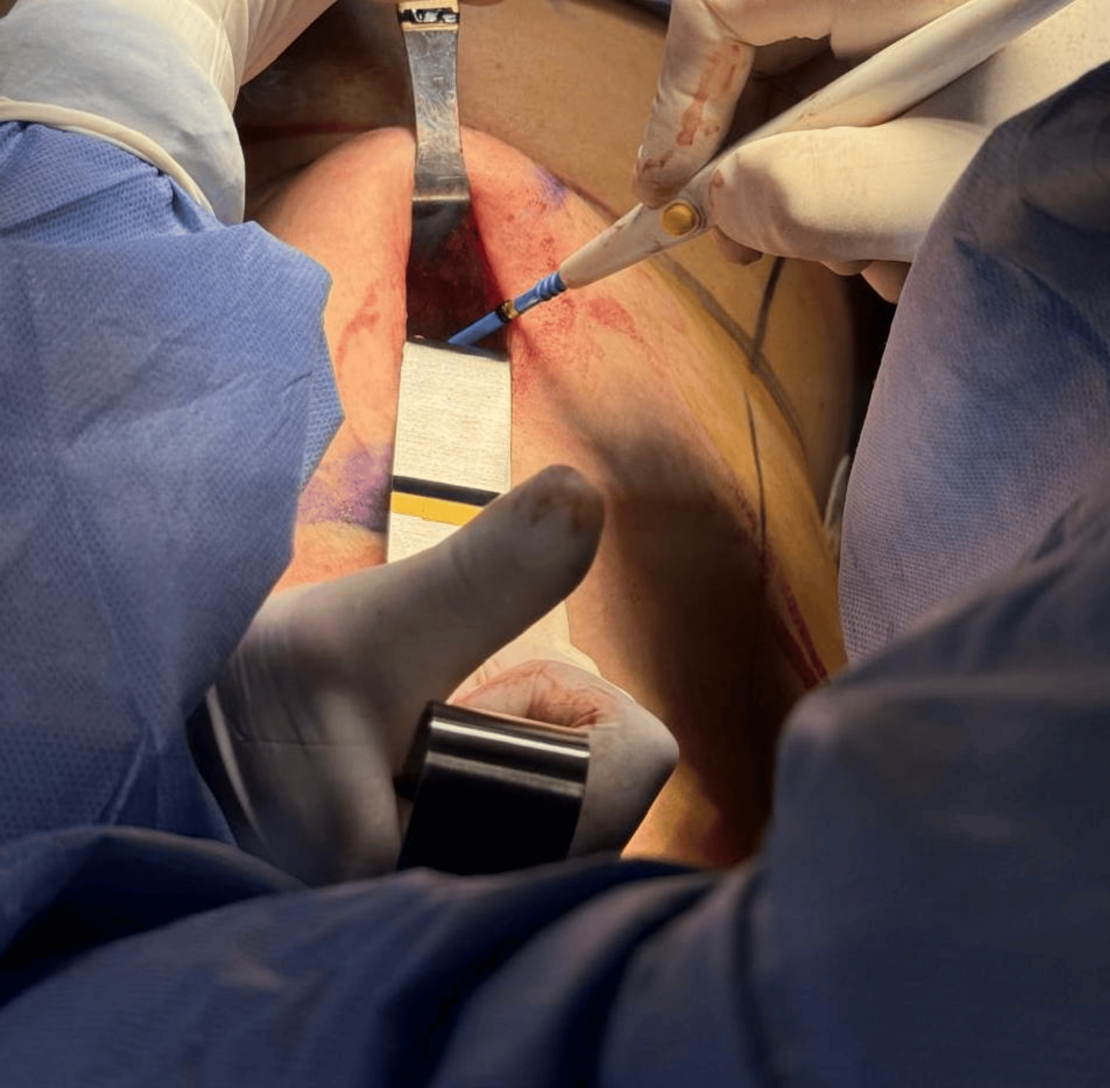
Incision in the mammary crease for exposure and removal of the left breast expander.

**Figure 2:**
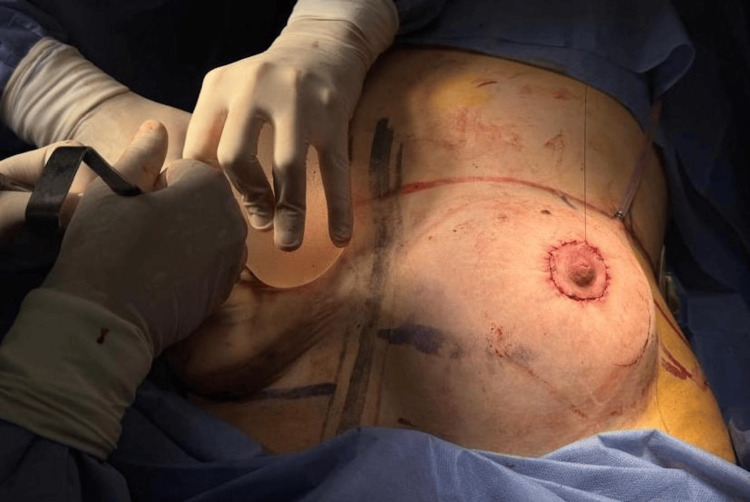
Placement of a 440 cc anatomical breast implant on the left breast.

For the definitive reconstructive procedure, the patient was admitted for removal of the left breast expander and placement of bilateral breast implants. During the procedure, an incision was made in the left inframammary fold, revealing the expander with free fluid inside (Figure 1). The prosthetic pocket was inspected, hemostasis confirmed, and a definitive 440 cc anatomical implant was placed in the left breast (Figure 2). Closure was performed in layers using 4-0 Monocryl for the deep dermis and 3-0 Monocryl for the superficial dermis.

**Figure 3:**
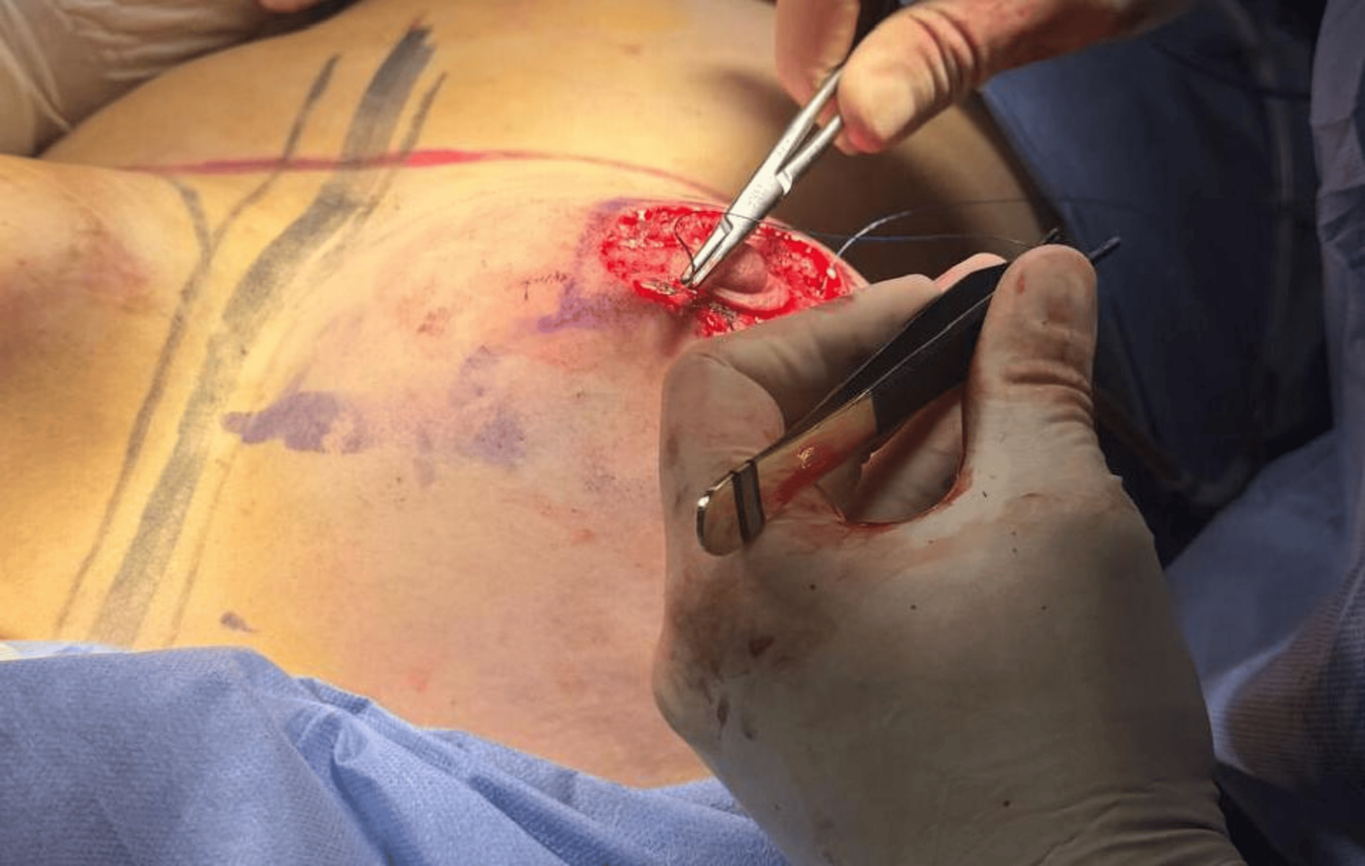
Remodeling of the right areola after placement of a definitive 300 cc anatomical implant.

**Figure 4:**
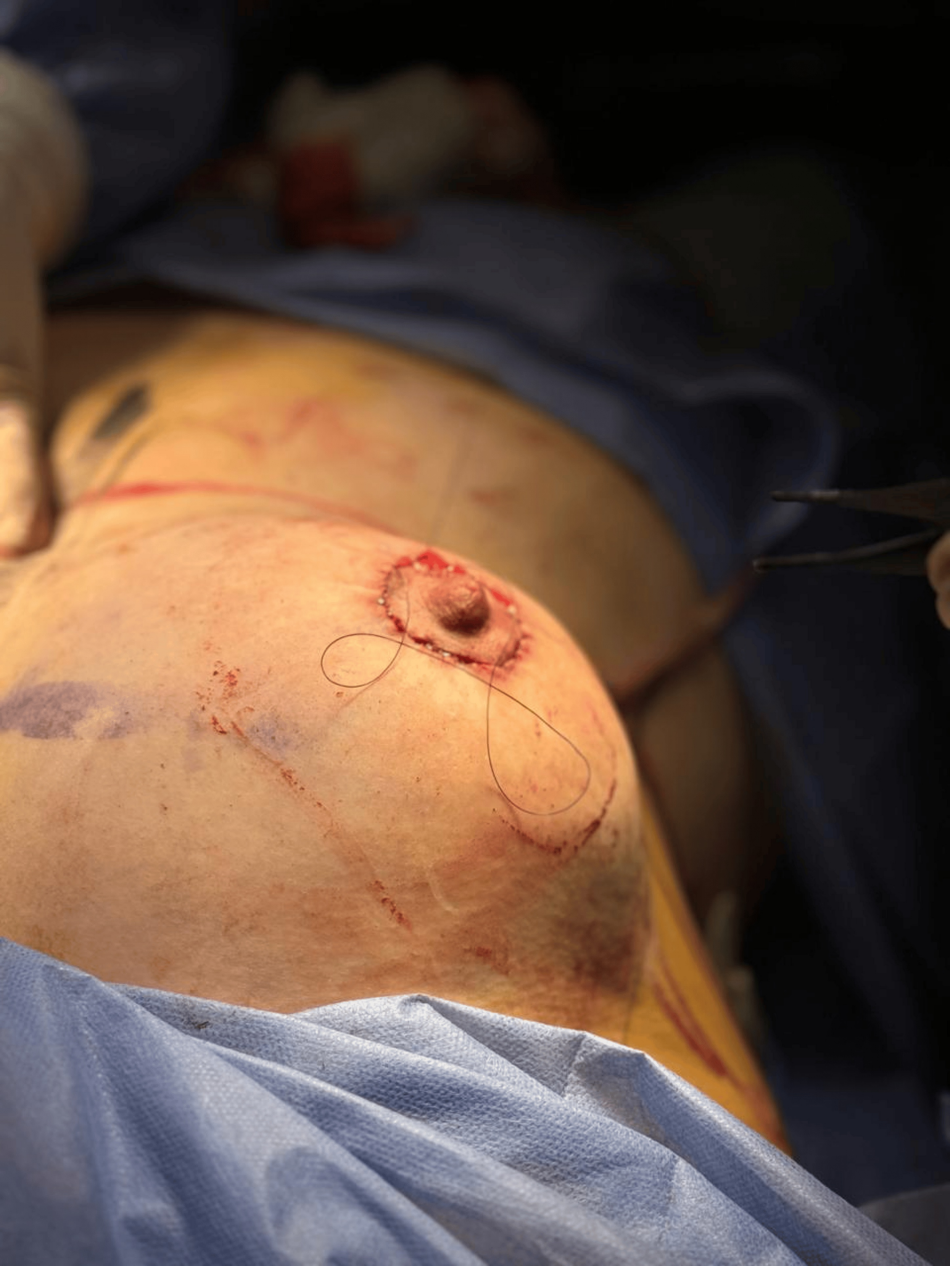
Right areola closure with 4-0 Monocryl.

**Figure 5:**
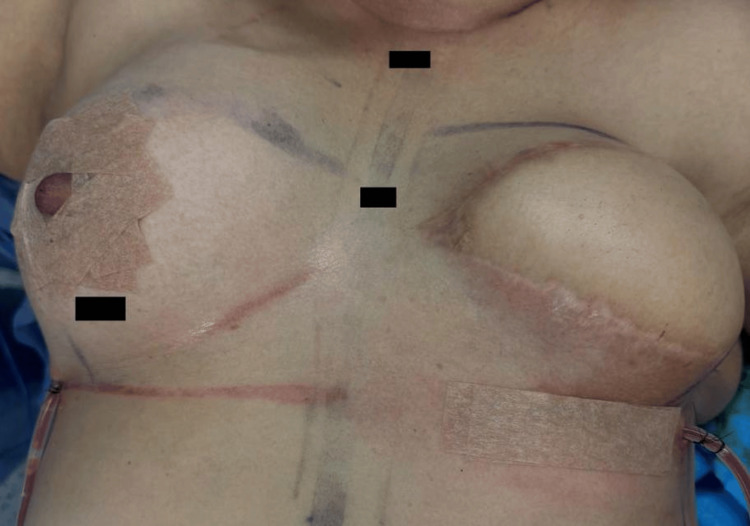
Immediate post-surgical evaluation.

Subsequently, the right breast was approached through a lower periareolar incision. A layered dissection was performed in the subcutaneous tissue for placement of a 300 cc anatomical implant for symmetrizing purposes (Figure 3). The procedure was completed with areolar remodeling and closure with 4-0 Monocryl (Figure 4). Finally, bilateral Blake-type drains were placed, secured with 3-0 Nylon (Figure 5).

In the immediate postoperative period, the patient progressed favorably, with no acute complications related to the procedure. She was discharged 24 hours later, with prescription painkillers and antibiotics, and a follow-up appointment was scheduled five days later.

## Discussion

Primary surgical management is indicated for patients with early breast cancer. This can include conservative surgery or total mastectomy, regardless of the surgical management of the axilla. It should be followed by adjuvant therapies as indicated [2]. Breast cancer affects one-seventh of women in the United States, making it the most common malignancy among women. Breast reconstruction is a continuously evolving entity that varies in complexity from the use of implants to autologous tissue [3].

Postmastectomy breast reconstruction has evolved significantly in recent decades, becoming an integral part of multidisciplinary breast cancer management. Current techniques not only aim to restore breast volume but also achieve satisfactory aesthetic results with the least possible functional impact. The choice between prosthetic or autologous reconstruction depends on multiple factors, including tumor characteristics, the indication for radiotherapy, the patient's body morphology, and personal preferences [4].

The incidence of breast cancer is high and also affects young women; this has determined a growing demand for increasingly demanding reconstructive procedures, reversing the historical and universal trend that mastectomized patients do not undergo reconstruction, overcoming the fear of surgical complications that may delay adjuvant treatment and the fear of masking a recurrence.

Reconstruction with tissue expanders followed by breast implants is one of the most widely used techniques due to its lower morbidity, shorter surgical times, and the possibility of being applied even in patients without sufficient autologous tissue. Trigos and Herrán reported that this technique provides predictable and aesthetically favorable results, especially when combined with fat grafts or acellular dermal matrices, which improve implant contour and coverage [5].

On the other hand, autologous reconstruction using musculocutaneous flaps such as TRAM, DIEP, or latissimus dorsi is considered ideal for patients who have received radiation therapy or for those who desire a more natural result. These techniques offer better radiation tolerance and a lower rate of complications such as capsular contracture. Bazualdo Fiorini et al. concluded that, although these options involve greater surgical complexity, they offer superior functional and aesthetic advantages in selected patients [6].

Currently, breast reconstruction is an integral part of breast cancer treatment, and patient selection is critical for successful outcomes. Stable, well-vascularized coverage is required to achieve acceptable and long-lasting results. Breast reconstruction with a tissue expander and implant is an attractive option for many women because of the absence of sequelae in the donor area, which are common with flap reconstructions, and because it facilitates a quick recovery. It is important that patients who are candidates for this type of reconstruction have undergone a prior diagnostic biopsy, which will determine the oncological criteria regarding the possibility of post-mastectomy radiation.

The indications for this type of breast reconstruction are sufficient, well-vascularized soft tissue to cover the expander, pectoral muscle preserved during the mastectomy, skin coverage that has not been previously irradiated and that will not be irradiated postoperatively, patient acceptance, and availability of frequent appointments to perform the tissue expansion procedure. Breast reconstruction with implants is contraindicated when skin coverage is inadequate, which may be secondary to previous biopsies or advanced local disease requiring extensive skin resection during mastectomy. Prior radiation therapy is a relative contraindication for implant reconstruction, as it presents a high rate of prosthetic exposure and capsular contracture [7].

Some authors consider immediate breast reconstruction preferable. However, for various reasons, delayed reconstruction is considered in patients who have already undergone a full therapeutic regimen (including possible radiation therapy), who have usually undergone mastectomies and axillary surgical procedures. These circumstances are not ideal from the plastic surgeon's perspective and can affect the surgical technique and compromise the final aesthetic result [8].

The current trend toward minimally invasive techniques has driven the adoption of the prepectoral approach, which positions the implant over the pectoral muscle, reducing postoperative pain and avoiding muscle animation. Tomita and Kubo noted that combining this technique with acellular dermal matrices results in a more anatomical reconstruction, with a lower risk of muscular complications and a shorter recovery period [9].

In patients with comorbidities or a history of radiation, mixed techniques integrating expanders, fat grafts, and local flaps have been explored. In a clinical series, Reynoso-Saldaña et al. documented complex cases resolved with individualized approaches, demonstrating that the versatility of these techniques allows them to be adapted even to adverse surgical conditions with successful results [10]. As reconstructive options continue to expand, particularly for patients with prior surgeries or complex anatomical considerations, recent evidence has demonstrated that even patients with a history of breast reduction can safely undergo oncoplastic reconstruction. Koenig et al. reported favorable outcomes in this population, highlighting the importance of individualized planning and the evolving safety of reconstructive techniques in challenging scenarios [11].

Despite technical advances that have contributed to improving both success rates and aesthetic outcomes, breast reconstruction continues to present significant challenges. Its approach requires comprehensive planning that considers medical, psychological, and social aspects. The involvement of multidisciplinary teams, long-term follow-up, and a patient-centered approach are essential pillars for providing truly comprehensive care.

## Conclusions

Breast reconstruction secondary to breast cancer is a fundamental part of treatment, as it not only restores the anatomy but also improves the psychological well-being and quality of life of patients. The technique with tissue expanders followed by implants remains a common option due to its relative simplicity and good aesthetic results, although it can present complications, especially in patients undergoing radiation therapy. The evolution of reconstructive surgery has allowed the use of autologous techniques such as TRAM, DIEP, or LD flaps, as well as hybrid methods with fat grafts and acellular dermal matrices, tailored to individual needs. The choice of technique should be based on a comprehensive evaluation and a multidisciplinary approach to achieve optimal results. Technological and surgical advances continue to expand the available options, offering safer and more satisfactory reconstructions for patients.
